# Case Report: MECP2 and SH3KBP1 variants associated with autism spectrum disorder and immune dysregulation

**DOI:** 10.3389/fimmu.2026.1814088

**Published:** 2026-06-15

**Authors:** Arnau Marin-Llobet, Cristina Hernando-Davalillo, Heidy Baide-Mairena, Pilar Llobet-Agullo, Neus Fornes Garcia, Veronica Perez-Herrera, Anna Baro-Serrano

**Affiliations:** 1 Pediatric Neurology Unit, Hospital General de Granollers, Granollers, Spain; 2 Department of Genetic Medicine–Instituto Pediátrico de Enfermedades Raras (IPER), Hospital Sant Joan de Deu, Barcelona, Spain; 3 Pediatric Allergy and Clinical Immunology Unit, Hospital General de Granollers, Granollers, Spain; 4 Pediatric Cardiology Unit, Hospital General de Granollers, Granollers, Spain

**Keywords:** autism spectrum disorder (ASD), chromosomal microarray (CMA) testing, immunodysregulation, inborn errors immunity, MECP2 duplication syndrome, neurodevelopmental disorders, *SH3KBP1*

## Abstract

Autism Spectrum Disorder (ASD) is a heterogeneous neurodevelopmental condition characterized by impairments in social communication and restricted, repetitive behaviors. Increasing evidence suggests that immune dysregulation may be present in a subset of individuals with neurodevelopmental disorders, although its role in ASD remains incompletely understood. We report two male patients with ASD, neurodevelopmental delay, immune dysregulation, and congenital cardiac anomalies. Genetic analysis using chromosomal microarray (CMA) identified distinct copy number variants on the X chromosome in each case. In patient 1, a duplication of the Xq28 region encompassing the *MECP2* gene was identified, consistent with MECP2 duplication syndrome, a well-established cause of neurodevelopmental impairment and recurrent infections. In this case, the clinical presentation was comparatively milder than typically described, highlighting phenotypic variability within this condition. In patient 2, a hemizygous deletion at Xp22.12 partially involving the *SH3KBP1* gene was detected and classified as a variant of uncertain significance (VUS). The patient presented with recurrent respiratory infections, impaired humoral immune responses, reduced B-cell counts, and neurodevelopmental impairment. This observation broadens the limited clinical spectrum associated with *SH3KBP1 v*ariants and may aid future assessment of their clinical relevance, although a causal relationship remains unproven. Together, these observations emphasize the value of comprehensive genetic testing in individuals with complex neurodevelopmental and systemic presentations and support consideration of immunological evaluation in selected patients with ASD who present with recurrent infections, allergic disease, or additional phenotypic features suggestive of inborn errors of immunity.

## Introduction

Neurodevelopmental disorders are a diverse group of conditions characterized by disruptions in nervous system development, resulting in impairments in cognition, behavior, and social functioning. Among these, Autism Spectrum Disorder (ASD) is a particularly complex and heterogeneous condition, defined by persistent deficits in social communication and interaction, along with restricted, repetitive behaviors and atypical sensory processing (1). Despite its clear diagnostic criteria, ASD exhibits considerable variability in clinical presentation and underlying etiology, complicating both diagnosis and management. Advances in diagnostic frameworks have revealed a rising prevalence, with ASD affecting approximately 1 in 36 children in the United States and 1–2% of children globally (2, 3). The disorder shows a notable male predominance (4:1 ratio of boys to girls) and a high heritability (70–90%), underscoring the significant contribution of genetic factors to its pathogenesis (4–8). Although ASD etiology remains incompletely understood, a complex interplay of genetic and environmental factors has been implicated, including pathogenic mutations, single nucleotide variants, copy number variations, advanced parental age, maternal health conditions, and fetal toxin exposure (9–11). More recently, growing evidence has implicated immune system dysfunction in the pathogenesis of ASD and other neurodevelopmental disorders (12–15). In this context, increasing attention has been directed toward the possibility that underlying immune abnormalities, including Inborn Errors of Immunity (IEI), may be present in a subset of individuals with ASD and could contribute to atypical clinical presentations (16).

IEI comprise more than 500 hereditary disorders that impair immune function and give rise to a broad spectrum of clinical manifestations involving nearly all medical specialties, including neurology, cardiology, pulmonology, gastroenterology, dermatology, and hematology (17, 18). These conditions are frequently associated with recurrent infections, autoimmunity, allergy, malignancy, and systemic inflammatory abnormalities. Neurological manifestations may constitute important clinical clues to the diagnosis of IEI, such as ataxia in ataxia-telangiectasia, tetraparesis in purine nucleoside phosphorylase deficiency, or herpes simplex encephalitis in Toll-like receptor 3 deficiency (19, 20). Despite this wide phenotypic spectrum, ASD has not traditionally been considered an indication for immunological evaluation and is rarely assessed (21, 22). Nevertheless, recent studies have suggested that immune dysregulation and IEI may be underrecognized in selected patients with ASD, particularly in those presenting with recurrent infections, autoimmunity, allergic manifestations, or additional systemic abnormalities (22–29). These observations support the need for greater awareness and consideration of immune system assessment in patients with ASD and atypical multisystemic features.

Here, we present two pediatric cases of ASD associated with immune dysregulation and congenital cardiac anomalies, illustrating a rare overlap between neurodevelopmental, immunological, and cardiovascular phenotypes. These cases highlight the importance of considering immunological evaluation in selected patients with ASD presenting with complex or syndromic features. In the first case, we identified a duplication involving the *MECP2* gene, consistent with MECP2 duplication syndrome, thereby expanding its recognized clinical spectrum. In the second case, we identified a variant in the *SH3KBP1* gene that may represent a potential contributor to both ASD and immune dysregulation.

## Case presentation

We present two male pediatric cases diagnosed with autism spectrum disorder (ASD) who also exhibit features of immune dysregulation. Written informed consent was obtained from the parents of each patient for participation in this study and for the publication of any potentially identifiable data included in this article. Ethical review and approval was not required in accordance with local legislation and institutional requirements.

Both cases demonstrate a complex interplay of neurodevelopmental delay, recurrent respiratory infections, and immunological abnormalities, underscoring the need for a multidisciplinary approach. Although they share overlapping features, including recurrent bronchitis and impaired vaccine responses, they differ in their genetic findings, cardiac anomalies, and clinical course. A detailed summary is provided in **Tables 1**–**3**.

**Table 1:** Clinical and family characteristics of the two patients.

Clinical characteristic	Patient 1	Patient 2
Sex/Age	Male, 15 years	Male, 11 years
Family history	Mother with type 1 diabetes mellitus; brother with bicuspid aortic valve	Mother with gestational diabetes mellitus; father with food allergy; sister with celiac disease
Perinatal history	Full-term birth; BW: 3800 g	Full-term birth; BW: 4170 g
Growth profile	Weight and height around the 50th percentile	Weight above the 99th percentile
Impaired social interaction	Onset at 2 years of age	Onset at 2 years of age
Cognitive difficulties	Onset at 3 years of age	Onset at 4 years of age
Emotional dysregulation	Not reported	Onset at 9 years of age
Cardiac abnormalities	Bicuspid aortic valve with severe stenosis, diagnosed at 6 years of age	Restrictive ventricular septal defect, diagnosed at birth
Obstructive bronchitis	Onset at 9 months of age	Onset at 5 months of age
Recurrent upper respiratory tract infections	Onset at 5 years of age	Onset at 4 years of age
Allergic diseases	House dust mite allergy, diagnosed at 10 years of age	*Alternaria alternata* allergy, diagnosed at 9 years of age
Other clinical conditions	Scheuermann kyphosis, diagnosed at 13 years of age	Pyogenic granuloma, diagnosed at 9 years of age

BW, birth weight; VSD, ventricular septal defect.

Data are presented as clinically relevant findings collected during longitudinal follow-up. Percentiles are expressed according to age- and sex-adjusted pediatric growth charts. *Alternaria alternata* is reported according to standard taxonomic nomenclature.

**Table 2:** Immunological and laboratory findings in both patients.

Laboratory parameter	Patient 1	Patient 2
IgG (mg/dL)	1332 (478.9–1433)	1386 (581.4–1652)
IgM (mg/dL)	134 (25.92–232.3)	**6** (47.41–251.8)
IgA (mg/dL)	111.2 (59.84–348.8)	68.8 (41.59–344.9)
IgE (IU/mL)	**1229** (0–100)	**226** (0–200)
IgG1 (mg/dL)	912 (325–894)	889 (315.6–1076)
IgG2 (mg/dL)	**69** (216–523)	175 (73–455)
IgG3 (mg/dL)	**30** (36–139)	80 (16–194)
IgG4 (mg/dL)	**4** (9–104)	**1** (1–153)
Lymphocyte subsets	Normal profile	Reduced CD19+ B cells
CD19+ cells (cells/µL)	347 (173–685)	**84** (276–640)
CD19+ cells (%)	19.3 (11.9–21.0)	**3.5** (12.0–24.0)
Anti–*Streptococcus pneumoniae* IgG (U/mL)	**>270** (53–79)	**5** (53–79)
Genetic findings	Partial duplication of *MECP2*, diagnosed at 6 years of age	Deletion at Xp22.12 including *SH3KBP1*, diagnosed at 10 years of age

Ig, immunoglobulin; IgE, immunoglobulin E; IgG, immunoglobulin G; IgM, immunoglobulin M; IgA, immunoglobulin A; CD, cluster of differentiation; µL, microliter; mL, milliliter.

Values in bold indicate results outside the corresponding reference ranges. Data are presented as absolute values with reference ranges in parentheses as 95% confidence intervals. Immunoglobulin subclasses and lymphocyte subsets were determined using standard clinical immunology laboratory methods (28). CD19+ values reflect circulating B lymphocyte populations (29). Age-specific reference ranges for CD19 + are presented as absolute cell counts (cells/µL) and percentages (%) of total lymphocytes (10th–90th percentiles). The “Anti–*Streptococcus pneumoniae* IgG” category includes responses to a mixture of the following serotypes: 1-5, 6B, 7F, 8, 9N, 9V, 10A, 11A, 12F, 14, 15B, 17F, 18C, 19F, 19A, 20, 22F, 23F, and 33F (30–32), measured as a single global titter. An impaired response— demonstrated by a failure to achieve a meaningful increase in pneumococcal-specific IgG after vaccination — is considered indicative of functional B cell antibody deficiency in patients with otherwise normal immunoglobulin levels and clinical features consistent with immunodeficiency (33).

Both patients received the 23-valent pneumococcal polysaccharide vaccine (Pneumovax 23^®^) and the 13-valent pneumococcal conjugate vaccine (Prevenar 13^®^) prior to this assessment. Pneumovax 23^®^ vaccine serotypes: 1, 2, 3, 4, 5, 6B, 7F, 8, 9N, 9V, 10A, 11A, 12F, 14, 15B, 17F, 18C, 19A, 19F, 20, 22F, 23F, and 33F; Prevenar 13^®^ vaccine serotypes: 3, 4, 6A, 6B, 7F, 9V, 14, 18C, 19A, 19F, and 23F. Source: Hospital Clínic de Barcelona (34).

**Table 3:** Treatment and clinical management of both patient.

Therapeutic intervention	Patient 1	Patient 2
Psychological and speech therapy	Initiated at 2 years of age	Initiated at 2 years of age
Antipsychotic (neuroleptic) treatment	Not prescribed	Initiated at 9 years of age
Bronchodilator therapy	Administered	Administered
Antibiotic therapy (short courses)	Administered	Administered
Inhaled corticosteroid therapy	Administered	Administered
Oral corticosteroid therapy (short courses)	Administered	Administered
Cardiac surgery	Surgical repair at 13 years of age	Not required

### Case 1

The first case involves a 15-year-old boy with a history of recurrent bronchitis since infancy and global developmental delay identified at two years of age. He was initially followed by a local Early Childhood Development Center (ECDC), receiving multidisciplinary support, including psychological and speech therapy. An autism spectrum disorder (ASD) diagnosis was established according to Diagnostic and Statistical Manual of Mental Disorders, Fifth Edition (DSM-5) criteria. Early signs suggestive of ASD were identified between 18 and 24 months of age at ECDC, where the child underwent assessment with the Autism Diagnostic Observation Schedule, Second Edition (ADOS-2). At five years of age, he was referred to our neurology clinic, where the diagnosis was confirmed using the same standardized tool in the context of persistent neurodevelopmental difficulties. During this evaluation, a cardiac murmur was detected, prompting referral to pediatric cardiology and genetic evaluation. Cardiology assessment confirmed a bicuspid aortic valve with severe stenosis. In patients presenting with congenital heart disease associated with neurodevelopmental delay, genetic testing is clinically indicated to investigate potential underlying syndromic disorders, including chromosomal abnormalities and conditions such as 22q11.2 deletion syndrome (35, 36).

Subsequent genetic studies using Multiplex Ligation-dependent Probe Amplification (MLPA) and array Comparative Genomic Hybridization (aCGH) identified a 680-kb duplication at chromosomal band Xq28 (arr[GRCh37] Xq28(152660683_153339015)x2), encompassing 26 protein-coding genes annotated in the NCBI Reference Sequence (RefSeq) database: PNMA6E, ZFP92, TREX2, HAUS7, BGN, ATP2B3, CCNQ, DUSP9, PNCK, SLC6A8, BCAP31, ABCD1, PLXNB3, SRPK3, IDH3G, SSR4, PDZD4, L1CAM, AVPR2, ARHGAP4, NAA10, RENBP, HCFC1, TMEM187, IRAK1, and MECP2 (NM_004992.4), including at least exons 3–4. Fluorescence *in situ* hybridization (FISH) studies confirmed a tandem duplication and gene expression studies showed *MECP2* RNA increased expression in peripheral blood. Unfortunately, confirmation in other tissues (such as skin biopsy) was not feasible due to their unavailability. Family studies revealed that the mother was asymptomatic except for type 1 diabetes mellitus, despite carrying the same partial duplication involving *MECP2* (MIM *300005). X-chromosome inactivation analysis using the human androgen receptor assay (HUMARA) demonstrated a completely skewed pattern (100:0) in peripheral blood. The variant has been deposited in the NCBI ClinVar database under accession number SCV005849919. The genes affected in each patient are listed in **Table 4** along with their corresponding SFARI gene score, an evolving database on genes implicated in autism susceptibility Genes are scored based on confidence: Category 1 (High Confidence), Category 2 (Strong Candidate), Category 3 (Suggestive Evidence), and S (Syndromic) (37).

**Table 4:** Genes affected in each patient and corresponding SFARI gene scores.

Gene	Patient 1 – SFARI score	Patient 2 – SFARI score
ZFP92	Not scored	—
PNMA6E	Not scored	—
TREX2	Not scored	—
HAUS7	Not scored	—
BGN	Not scored	—
ATP2B3	Not scored	—
CCNQ	Not scored	—
DUSP9	Not scored	—
PNCK	Not scored	—
SLC6A8	2	—
BCAP31	Not scored	—
ABCD1	Not scored	—
PLXNB3	Not scored	—
SRPK3	Not scored	—
IDH3G	Not scored	—
SSR4	3	—
PDZD4	Not scored	—
L1CAM	Not scored	—
AVPR2	Not scored	—
ARHGAP4	Not scored	—
NAA10	3S	—
RENBP	Not scored	—
HCFC1	S	—
TMEM187	Not scored	—
IRAK1	Not scored	—
MECP2	1S	—
SH3KBP1	—	Not scored

SFARI, Simons Foundation Autism Research Initiative.

SFARI gene scores classify genes according to evidence for association with autism spectrum disorder: Category 1 (high-confidence genes), Category 2 (strong candidate genes), Category 3 (suggestive evidence), and S (syndromic genes). The symbol “—” indicates that the gene was not identified or not applicable in the corresponding patient. Gene annotations are based on the SFARI Gene database at the time of analysis (https://gene.sfari.org/database/human-gene/, accessed April 5th 2026).

Given the combination of neurodevelopmental delay, congenital heart disease, and recurrent bronchitis, the patient was referred for immunology and allergy evaluation. Immunologic studies demonstrated elevated total Inmunoglobulin (Ig) E, with high specific Ig E to house dust mite allergens, a selective IgG2 deficiency, and a poor vaccine response. Total IgG and IgG1 levels remained slightly elevated for the patient’s age, while IgG2 was significantly reduced and other IgG subclasses were below the lower limit of normal. Although low IgG4 levels are not always clinically relevant, as many healthy individuals have low or undetectable IgG4, the concurrent reduction in other subclasses (such as IgG2 and IgG3) may be clinically significant and warrants continued monitoring. Despite receiving multiple booster doses of tetanus and pneumococcal vaccines (Pneumovax 23^®^ at 8 years of age and Prevenar 13^®^ at 9 years), his immunological response remained suboptimal during follow-up from 7 to 14 years old. While protective thresholds and serotype-specific criteria vary across guidelines (31, 32), the low global pneumococcal IgG titer observed after Pneumovax vaccination is consistent with an impaired humoral response and suggests a B-cell functional defect. However, he showed adequate responses to measles and varicella vaccines, and an improved response following a second dose of Pneumovax 23^®^ administered at age 15 (**Table 2**). Lymphocyte subpopulations and other immunoglobulin levels were normal, and secondary causes of immunodeficiency were excluded. Currently, he does not meet the diagnostic criteria for IgG subclass deficiency due to the absence of clinically significant infections (33). At 13 years old, he underwent successful valvuloplasty and has since remained asymptomatic from a cardiac perspective. Currently, he is under multidisciplinary follow-up, including educational adaptations, low-dose inhaled budesonide, annual influenza vaccination, and antihistamines. Given the absence of significant infections and well-controlled obstructive bronchitis, immunoglobulin replacement therapy has not been initiated.

### Case 2

**Figure 1:**
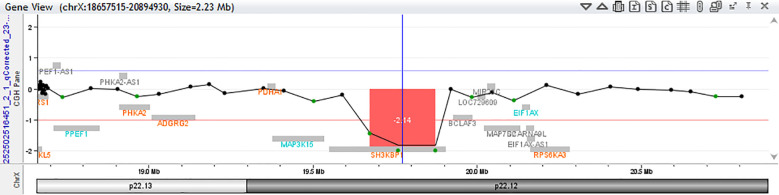
Genomic view of an interval on chromosome X (chrX: 18,657,515–20,894,930; 2.23 Mb) showing copy number variations (CNVs) in case 2. Footnotes: The x-axis represents genomic coordinates and the y-axis shows normalized log2 copy number ratios. Black dots connected by lines indicate segmental copy number values. Neutral regions are shown in black, and copy number losses in green. The red-shaded region indicates a significant loss (log2 ratio < −2), consistent with a hemizygous deletion. Genes within the affected interval include *PEPT4-AS1, PHKA2-AS1, MAP3K16*, and *SH3KBP1*. Cytogenetic bands (p22.13 and p22.12) are indicated below the plot.

The second case is an 11-year-old boy who has been followed by cardiology specialists since four months of age due to a restrictive ventricular septal defect (VSD). He has remained asymptomatic from a cardiac perspective. His medical history is notable for recurrent bronchitis since infancy and developmental delay identified at two years of age, when speech therapy was initiated at the Early Childhood Development Center (ECDC). At that time, features suggestive of ASD were also recognized. Autism diagnoses was established at 24 months of age according to the DSM-5 criteria. The assessments were conducted using the Autism Diagnostic Observation Schedule, Second Edition (ADOS-2), administered by trained professionals at ECDC. At three years of age, the patient was referred to gastroenterology due to a family history of celiac disease in his sister, despite presenting no gastrointestinal symptoms; however, celiac disease was subsequently ruled out. His weight and height have consistently exceeded the 99th percentile, with a body mass index (BMI) greater than 2 standard deviations (SD) above the mean since the age of five. At six years old, the diagnosis of autism spectrum disorder (ASD) was confirmed at an external center, and treatment was initiated with atomoxetine and risperidone. At seven years old, he was referred to immunology and allergy services due to recurrent episodes of bronchitis. Initial immunological workup revealed immunoglobulin M (IgM) levels below 20 mg/dL, elevated IgE levels without sensitization to common aeroallergens, absent serologic responses to measles (after two vaccine doses), varicella (after two vaccine doses), and pneumococcal conjugate vaccines (following four doses of PCV13 administered during infancy. Follow-up between the ages of 7 and 11 years demonstrated persistently low IgM levels (ranging between 5 and 6 mg/dL). Initially, at 8 years old, he showed a satisfactory response to pneumococcal vaccination (one dose of Pneumovax 23^®^), but this response diminished over time, and he did not respond to a second Pneumovax 23^®^ dose at age 11. At nine years old, an additional varicella vaccine dose elicited a good immune response, whereas an additional measles vaccine dose did not. Analysis of lymphocyte subpopulations consistently demonstrated low B-cell counts for his age (87 cells/µL, 3.2% of total lymphocytes at 8 years old; 84 cells/µL, 3.5% of total lymphocytes at 11 years old), while other lymphocyte subsets remained within normal ranges. At the age of 9 years, he developed allergic rhinitis and asthma, with confirmed sensitization to *Alternaria alternata* via skin prick testing and specific IgE. His symptoms are currently well-controlled with antihistamines and inhaled corticosteroids and long-acting beta-2 agonists (ICS-LABA). Despite his immunologic abnormalities and poor vaccine responses, immunoglobulin replacement therapy has not been initiated due to the absence of significant infections (32, 33). At 10 years of age, the patient was referred to our Neurology Clinic for evaluation of autism spectrum disorder (ASD) and borderline intellectual disability. Genetic testing using array comparative genomic hybridization (aCGH) identified a hemizygous interstitial deletion on the short arm of the X chromosome (arr[GRCh37] Xp22.12(19676448_19875998)x0),partially involving the *SH3KBP1* gene (OMIM *300374; CIN85). The identified deletion in our patient spans approximately 200 kb and is predicted to disrupt exons 2–6, or potentially a larger region encompassing up to exons 1–12, including the 5′UTR region, as illustrated in **Figure 1**. This alteration is expected to result in loss of normal *SH3KBP1* protein function. Importantly, the deletion occurred *de novo*, as confirmed by its absence in the patient’s mother. The variant has been deposited in the NCBI ClinVar database under accession number SCV005849918. This variant is classified as a variant of uncertain significance (VUS) due to the limited number of reported cases involving this gene in neurodevelopmental and immunological phenotypes. No additional genomic studies were performed, as the identified finding was considered consistent with the clinical presentation.

## Discussion

We report two children with autism spectrum disorder (ASD) and multisystem involvement who exhibited partially overlapping clinical features despite distinct underlying genetic alterations. Both cases involved ASD, intellectual disability, congenital heart disease, recurrent bronchitis, and allergies. Initial clinical evaluations focused on ASD and congenital heart disease, ultimately leading to referral to pediatric clinical immunology for further work-up. Immune abnormalities were confirmed in both cases, and genetic testing identified distinct X-linked variants classified as likely pathogenic and of uncertain significance, respectively, further supporting their syndromic presentations.

Case 1 was diagnosed with MECP2 duplication syndrome, an X-linked disorder predominantly affecting males and characterized by developmental delay, intellectual disability, infantile hypotonia, motor and speech impairment, recurrent infections, seizures, and gastrointestinal dysfunction (38–40). Female carriers were traditionally considered asymptomatic because of skewed X chromosome inactivation (XCI) favoring the normal allele; however, symptomatic female carriers have increasingly been recognized, including individuals with neuropsychiatric manifestations, learning disabilities, and autoimmune or endocrine disorders (41–43). The *MECP2* gene encodes a protein essential for normal neuronal function, and increased dosage results in protein overexpression associated with neurodevelopmental and immunological manifestations. Our patient presented with a relatively mild phenotype including intellectual disability, hypotonia, allergy, recurrent respiratory infections, reduced immunoglobulin levels, and suboptimal vaccine responses. Because congenital heart disease is not typically associated with MECP2 duplication syndrome, alternative syndromic diagnoses such as velocardiofacial syndrome and 22q11.2 deletion syndrome were initially considered (35, 36). Although these conditions were excluded by array CGH, the diagnostic evaluation ultimately led to the identification of the Xq28 duplication involving *MECP2*. Interestingly, a bicuspid aortic valve was also identified in the patient’s 4-year-old brother, who did not have ASD and tested negative for the familial *MECP2* duplication, suggesting that the cardiac anomaly may be unrelated to the duplication itself.

Notably, the other genes included in the duplicated region are not currently classified as triplosensitive according to the ClinGen Dosage Sensitivity database (44), although their contribution cannot be completely excluded. Previous studies have shown that duplication size correlates with phenotypic variability (42) and data from the Hospital Sant Joan de Déu cohort further support a central role for *MECP2* and *IRAK1* dosage in determining the clinical phenotype (43). The absence of *RAB39B*, a gene associated with more severe neurodevelopmental manifestations, may partially explain the relatively mild presentation observed in our patient. In addition, *IRAK1*, which is commonly co-duplicated with *MECP2*, has been implicated in immune dysregulation and susceptibility to recurrent respiratory infections (44). Altered IgG subclass profiles and dysregulated inflammatory responses have also been reported in MECP2 duplication syndrome, although the precise immunological mechanisms remain incompletely understood (45, 46). Overall, these findings support the hypothesis that the phenotype observed in patient 1 is primarily related to *MECP2* and *IRAK1* duplication. Regardless of the underlying mechanisms, this case highlights the importance of immunological evaluation in patients with MECP2 duplication syndrome, as immune abnormalities may contribute substantially to morbidity and influence clinical management.

Case 2 involved an 11-year-old male with ASD, recurrent respiratory infections since infancy, asthma, allergies, obesity, and congenital heart disease. Genetic analysis identified a hemizygous Xp22.12 deletion partially involving *SH3KBP1*. Although the clinical significance of this variant remains uncertain, the phenotype observed in our patient raises the possibility that partial *SH3KBP1* deletion may contribute to combined neurodevelopmental and immunological abnormalities. The *SH3KBP1* gene encodes CIN85, an adaptor protein involved in immune receptor signaling and B-cell receptor (BCR)-mediated activation, processes essential for normal humoral immunity and B-cell differentiation. Consistent with this role, *SH3KBP1* deficiency, currently classified as Immunodeficiency 61 (IMD61), has recently been associated with impaired BCR signaling, defective antibody production, altered memory B-cell subsets, and susceptibility to recurrent bacterial infections (47–50). To date, only two affected individuals have been reported in the literature. Keller et al. (50) described two brothers with X-linked recessive immunodeficiency caused by loss-of-function variants in *SH3KBP1*. In addition to humoral immune abnormalities, both patients also presented with neurodevelopmental manifestations, including attention-deficit/hyperactivity disorder (ADHD) and mild cognitive impairment. The overlap between the phenotype reported by Keller et al. and the clinical manifestations observed in our patient supports a possible association between *SH3KBP1* dysfunction and combined immune and neurodevelopmental abnormalities. In addition, *SH3KBP1* deletions and related copy-number variants involving neurodevelopmental phenotypes have also been reported across genomic databases and X-linked intellectual disability cohorts (51, 52), further supporting a potential role of this gene in neurodevelopmental processes. The finding of congenital heart disease, not previously reported in IMD61, may suggest a broader clinical spectrum; however, this association remains speculative, as a causal relationship between *SH3KBP1* alterations and congenital heart defects has not been established. Collectively, several observations support the potential clinical relevance of the variant identified in our patient, including its *de novo* occurrence, the predicted loss-of-function effect associated with the partial deletion, and the phenotypic overlap with previously reported IMD61 cases. These findings raise the possibility that the current classification of the variant as of uncertain significance may warrant future reassessment. However, the available evidence is still insufficient to establish pathogenicity conclusively (53), and further functional studies are required.

These findings emphasize the importance of genetic testing in patients with complex neurological, immunological, and multisystem presentations, as it may provide valuable insights into the underlying etiology and guide clinical management. Inborn errors of immunity (IEI) are increasingly recognized as multisystem disorders that may include neurodevelopmental manifestations in addition to recurrent infections and immune dysregulation (18–20); however, they remain frequently underdiagnosed because of their heterogeneous clinical presentation (54). This is particularly relevant in individuals with ASD, especially those with more severe phenotypes, in whom medical evaluations may be limited by the challenges associated with procedures such as blood tests or hospital visits. Consequently, immunodeficiency and other comorbidities may remain underrecognized, potentially contributing to worse clinical outcomes and increased premature mortality, as previously highlighted by O’Nions et al. (55). Recognition of immune abnormalities in children with ASD and multisystem involvement may therefore facilitate earlier genetic diagnosis and a more comprehensive clinical evaluation. Our report further supports the importance of immunological assessment in selected patients with ASD, particularly when recurrent infections or additional systemic manifestations are present (16).

The growing recognition of interactions between immune dysregulation and ASD also raises the possibility of exploring novel therapeutic approaches (56). Isolated reports have described improvements in behavioral symptoms, irritability, hyperactivity, social withdrawal, and seizures following immunoglobulin administration in selected patients with ASD; however, current evidence remains limited and insufficient to support broad clinical recommendations (57). In our patients, management consisted of symptomatic neurological treatment together with pneumococcal vaccination and preventive therapy for asthma and allergic rhinitis. Immunoglobulin replacement therapy and biological agents were not indicated, as neither patient met the clinical or immunological criteria for these treatments. Further studies are needed to determine whether more targeted immunological interventions could improve neurological or systemic outcomes in children with ASD and associated immune abnormalities.

## Limitations

This case report has several limitations. First, functional studies were not performed to directly assess the biological impact of the identified genetic variants, limiting definitive conclusions regarding their pathogenicity. Second, although both variants were located on the X chromosome and were compatible with X-linked inheritance, segregation analysis was limited to maternal testing, and extended family studies were unavailable, precluding a more comprehensive assessment of inheritance patterns and possible mosaicism. Third, while the clinical relevance of *MECP2* dosage alterations is well established, the number of reported cases involving *SH3KBP1* deficiency remains extremely limited, restricting robust genotype–phenotype correlations for this gene. In addition, whole-exome sequencing (WES) and whole-genome sequencing (WGS) were not available in either patient and may therefore have limited the identification of additional contributing variants. Finally, although the clinical and immunological findings support a potential association between the identified variants and the observed phenotypes, the current evidence remains insufficient to establish definitive causality.

## Conclusions

Both cases presented in this report broaden the recognized phenotypic spectrum of genetic disorders associated with autism spectrum disorder (ASD) and immune dysfunction. In Case 1, MECP2 duplication syndrome was associated with a relatively mild neurodevelopmental presentation accompanied by immune abnormalities, without the severe infectious phenotype commonly described. In Case 2, a hemizygous deletion involving *SH3KBP1* was identified in a patient with neurodevelopmental impairment, immune dysregulation, and congenital heart disease, adding to the limited number of reported cases involving this gene.

Although *SH3KBP1* has been implicated in both immunodeficiency and neurodevelopmental phenotypes, current evidence remains limited. Our findings support a possible contribution of *SH3KBP1* to shared immunological and neurodevelopmental pathways; however, its pathogenic role has not yet been definitively established. The presence of congenital heart disease in our patient may also suggest a broader phenotypic presentation associated with *SH3KBP1* alterations, although additional evidence is needed to clarify this relationship.

Collectively, these cases highlight the importance of considering genetic testing in patients with complex neurodevelopmental and systemic manifestations, as it may uncover disorders affecting shared immune–neurodevelopmental pathways. Nevertheless, definitive genotype–phenotype correlations remain to be established, and additional functional and clinical studies are necessary to better define the biological role of *MECP2* and *SH3KBP1.*

These findings also underscore the potential value of immunological evaluation in selected patients with ASD who present with recurrent infections, allergic disease, or other systemic manifestations. In such cases, a multidisciplinary approach may facilitate earlier recognition of underlying genetic and immune abnormalities, potentially improving diagnostic accuracy and clinical management.

## Data Availability

The raw data supporting the conclusions of this article will be made available by the authors upon reasonable request. The datasets generated and analyzed during the current study are available in the ClinVar repository, (https://www.ncbi.nlm.nih.gov/clinvar/) under accession numbers SCV005849919 (case 1) and SCV005849918 (case 2).
